# Efficacy of the Stent‐in‐Stent Technique as a Rescue Method for Removing Embedded Metallic Biliary Stents

**DOI:** 10.1002/deo2.70246

**Published:** 2025-12-17

**Authors:** Yasuhiro Komori, Susumu Hijioka, Yoshikuni Nagashio, Shota Harai, Daiki Yamashige, Kazunori Onuma, Keita Fujisaki, Daiki Agarie, Kohei Okamoto, Shin Yagi, Soma Fukuda, Masaru Kuwada, Joshua Josef Torres, Keijiro Ueda, Nao Fujimori, Yutaka Saito, Yoshihiro Ogawa, Takuji Okusaka

**Affiliations:** ^1^ Department of Hepatobiliary and Pancreatic Oncology National Cancer Center Hospital Tokyo Japan; ^2^ Department of Medicine and Bioregulatory Science Graduate School of Medical Sciences Kyushu University Fukuoka Japan; ^3^ Endoscopy Division National Cancer Center Hospital Tokyo Japan

**Keywords:** biliary tract neoplasms, endoscopic retrograde cholangiopancreatography, hepaticojejunostomy anastomotic stricture, pancreatic neoplasm, self‐expanding metallic stent

## Abstract

**Objectives:**

Self‐expandable metal stents (SEMS) may become embedded because of tissue hyperplasia or tumor ingrowth, making their removal challenging. The stent‐in‐stent (SIS) method, which involves placing another SEMS inside to compress the tissue and aid removal, is a known rescue approach for stent removal. However, its efficacy across anatomical routes, optimal timing of removal, and predictors of difficult stent removal remain unclear.

**Methods:**

We retrospectively reviewed 17 patients treated between April 2018 and May 2025. Embedded stents were placed via the transpapillary route, endoscopic ultrasonography‐guided hepaticogastrostomy (EUS‐HGS), or choledochojejunal anastomosis. Technical success rates and adverse events were evaluated.

**Results:**

The overall technical success rate was 76.5%, with rates of 80.0%, 100%, and 33.3% for the transpapillary route, EUS‐HGS, and choledochojejunal anastomosis, respectively. Three of the four failures occurred when removal was attempted within 4 weeks of the second stent placement. Stent removal was successful in 100% of the hyperplasia cases but in 60% of the ingrowth cases. Cholecystitis occurred in one case after the second stent placement.

**Conclusions:**

The SIS method is feasible for the transpapillary route and EUS‐HGS but may show limited efficacy in choledochojejunal anastomosis. Stent removal 4 weeks after the second stent placement improves the success rate. The SIS method may be less effective in cases of malignant ingrowth.

## Graphical abstract

The stent‐in‐stent technique involves placing another fully covered metal stent inside an irremovable embedded stent to compress tissue and enable safe removal. In this study of 17 patients, the overall technical success rate was 76.5%, with higher efficacy via EUS‐guided hepaticogastrostomy (100%) and transpapillary routes (80%). Delaying removal for over 4 weeks has the potential to improve outcomes, and the effectiveness may be lower in cases with malignant ingrowth (60%) or choledochojejunal anastomosis (33.3%).

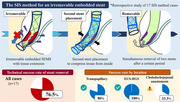

## 1 Introduction

Self‐expandable metal stents (SEMS) are widely used to provide favorable patency periods for malignant biliary strictures [1, 2]. However, long‐term SEMS placement can lead to being embedded by tumor ingrowth or mucosal hyperplasia, making stent removal difficult [3]. Conventional methods, such as grasping forceps or snares, may be ineffective for removing embedded stents [4]. In particular, the endoscopic removal of uncovered SEMS (UCSEMS) has shown a considerably lower success rate than that of fully covered SEMS (FCSEMS), ranging from 0% to 38.4% [5, 6]. In cases where stent removal is difficult using conventional methods, the stent‐in‐stent (SIS) method can serve as a rescue technique.

The SIS method was first reported in 2010 as a technique for removing embedded stents from the bile duct [7]. The SIS method involves placing an FCSEMS inside the lumen of an embedded stent, compressing the tissue that has expanded into the stent lumen, and subsequently removing both stents. In cases of malignant biliary obstruction and limited life expectancy, stent removal is not always necessary if a second stent is placed within the lumen of the first stent to provide adequate biliary drainage. However, in cases where chemotherapy has improved malignant strictures and biliary stenting is no longer required or in patients with benign strictures, stent removal can achieve a stent‐free status. In cases where long‐term stent placement has resulted in the accumulation of a large amount of biliary sludge or stones, stent removal and bile duct cleaning may help prolong stent patency. Furthermore, in cases with an expected favorable long‐term prognosis, the removal of embedded SEMS may be considered appropriate.

The SIS method was originally developed for embedded esophageal stents, and several case series have demonstrated its usefulness in esophageal stenting [8, 9]. Although several reports have described the application of the SIS method to embedded biliary stents [7, 10, 11, 12, 13, 14, 15, 16, 17], no case series involving more than five patients has assessed the efficacy of the SIS method. Therefore, the optimal timing for stent removal and factors contributing to the difficulty of stent removal have not been well established. Furthermore, most of these reports involved patients with a normal anatomy who underwent biliary SEMS removal via the transpapillary route [18]. Consequently, the efficacy and safety of the SIS method in alternative clinical settings, such as endoscopic ultrasound‐guided biliary drainage (EUS‐BD) or in individuals with choledochojejunal anastomosis, remain uncertain.

In this study, we conducted a retrospective analysis of cases in which the SIS method was used to remove embedded biliary SEMS. These included cases of transpapillary stent placement and those in which the stents were placed via EUS‐guided hepaticogastrostomy (EUS‐HGS) or choledochojejunal anastomosis.

## 2 Methods

### 2.1 Patients

We retrospectively identified cases in which the SIS method was used to remove embedded SEMS at the National Cancer Center Hospital, Japan, between April 2018 and May 2025. Cases in which the first stent was unintentionally removed along with the second stent during its removal were excluded. This study was conducted in accordance with the principles of the Declaration of Helsinki and approved by the Ethics Committee of the National Cancer Center Hospital, Japan (Ethical Approval Number: 2018‐149).

### 2.2 Techniques

**FIGURE 1:**
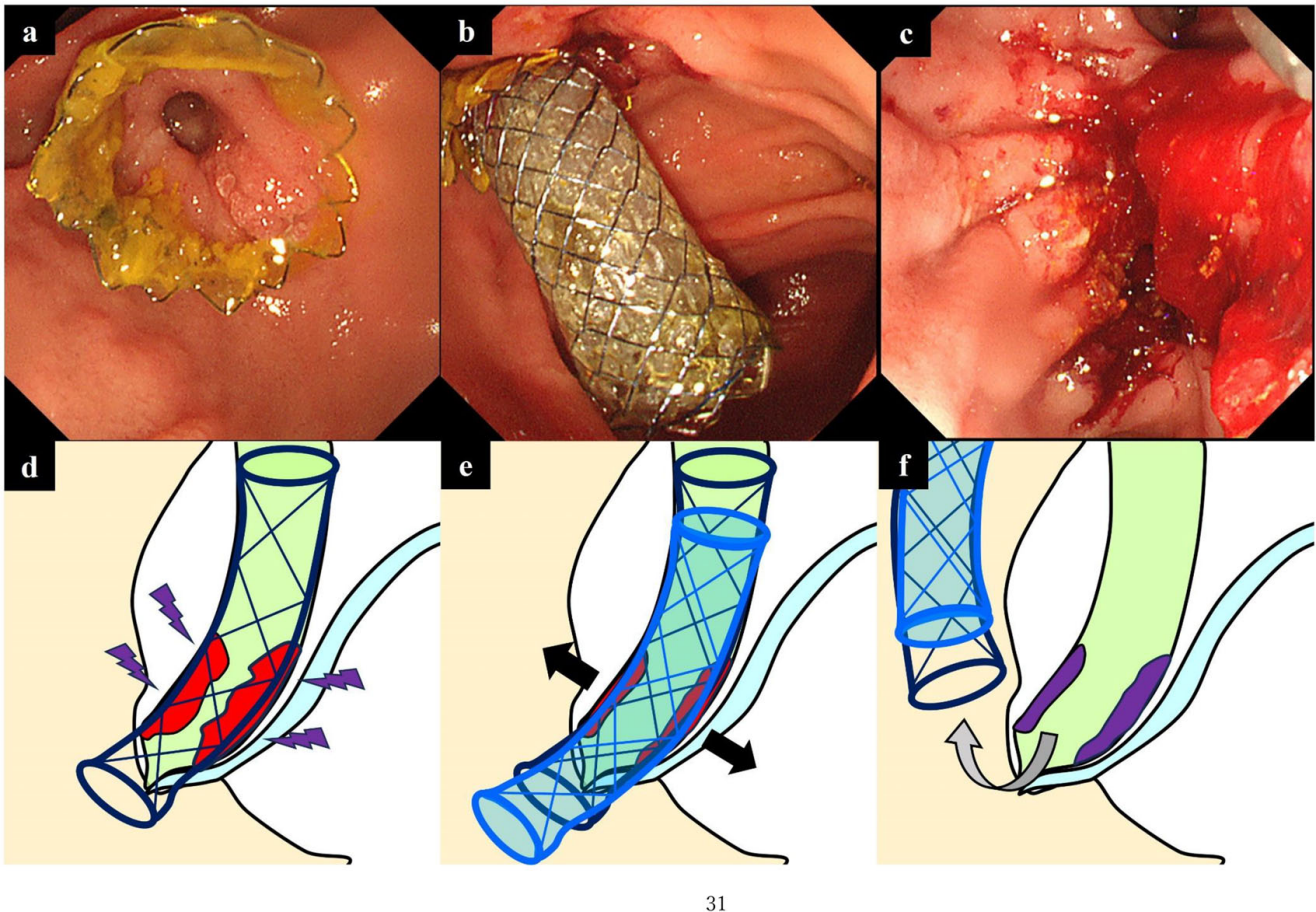
Standard stent‐in‐stent method for removal of embedded stents. (a, d) Hyperplasia or the tumor itself has expanded into the stent lumen through the mesh, causing the stent to become embedded and difficult to remove (embedded stent). (b, e) The radial force of another covered stent placed inside the lumen of the embedded stent compresses the hyperplastic tissue outward (stent‐in‐stent method). (c, f) After a certain period, the tissue that has expanded into the stent lumen is compressed using the stent‐in‐stent method, allowing removal of both stents.

A duodenoscope (TJF‐260V, TJF‐Q290V; Olympus, Tokyo, Japan) and a double‐balloon endoscope (EI‐580BT; FUJIFILM Medical, Tokyo, Japan) were used. An FCSEMS (second stent) was placed inside the embedded SEMS (first stent) lumen, which could not be removed using conventional methods, such as grasping forceps and snares, owing to tissue ingrowth or hyperplasia between the stent mesh. The radial force of the second stent compresses the tissue within the first stent, thereby aiding stent removal (Figure 1). The second stent was deployed to overlay all sites of tissue expansion within the first stent (Figure S1). The two stents were removed endoscopically using grasping forceps or snares after a set period following the second stent placement (Video S1). The selection of the stent type and timing of attempted removal after the second stent placement, within the embedded stent, were left to the discretion of the endoscopist.

### 2.3 Definitions of Outcomes

Regarding the etiology of stent embedding, cases with benign strictures and those with malignant strictures in which stent narrowing occurred at a site distant from the tumor were defined as stent embedding due to hyperplasia. All other cases of malignant tumor‐related stent embedding were defined as ingrowth. Technical success was defined as the removal of both SEMS in the first session. The procedure was considered technically unsuccessful if removal was completed in more than one session. Adverse events were assessed using the AGREE classification [19]. The primary outcome was the technical success rate of all embedded stents. In addition, we evaluated the technical success rate of stents placed via the transpapillary route, EUS‐HGS, and choledochojejunal anastomosis; the characteristics of the second stent; and the incidence of adverse events as secondary outcomes. We also evaluated the duration between the first and second stent placements using the SIS method, as well as the interval from the second stent placement to the attempted removal.

### 2.4 Statistical Analysis

Continuous variables, including age and stent dwell time, are presented as medians and ranges, and categorical variables are expressed as numbers and proportions. All statistical analyses were performed using JMP, version 18.0 (SAS Institute Inc., Cary, NC, USA).

## 3 Results

### 3.1 Patient Characteristics

In this study, we attempted to remove 17 embedded stents using the SIS method. The baseline patient characteristics are summarized in Table 1. The median age was 64 years (range, 36–82 years), and nine patients (52.9%) were male. The underlying diseases included pancreatic cancer in six cases (35.3%), biliary tract cancer in six cases (35.3%), pancreatic neuroendocrine tumor in four cases (23.5%), and common bile duct stones in one case (5.9%). The placement locations were transpapillary in 10 cases (58.8%), EUS‐HGS in four cases (23.5%), and choledochojejunal anastomosis in three cases (17.7%). Regarding anatomical status, 11 patients (64.7%) had normal anatomy, and six patients (35.3%) had surgically altered anatomy. The reasons for stricture at the stent placement site were malignancy in 14 cases (82.3%) and benignity in three cases (17.7%). The etiology of SEMS embedding was ingrowth in 10 cases (58.8%) and hyperplasia in seven cases (41.2%). The indications for stent removal were as follows: anticipated long‐term survival (46.9%, eight cases), improvement of biliary stricture (17.7%, three cases), stenting for benign biliary stricture (17.7%, three cases), extensive bile duct stone formation (11.8%, two cases), stent‐induced cholecystitis (5.9%, one case), and cholecystitis (7.7%, one case).

**TABLE 1:** Patient characteristics.

	*n* = 17
Age, years	64 (36–82)
Sex, male	9 (52.9%)
Primary disease	
Pancreatic cancer	6 (35.3%)
Biliary tract cancer	6 (35.3%)
Pancreatic neuroendocrine tumor	4 (23.5%)
Bile duct stone	1 (5.9%)
Stent location	
Transpapillary	10 (58.8%)
EUS‐HGS	4 (23.5%)
Choledochojejunal anastomosis	3 (17.7%)
Anatomy	
Normal	11 (64.7%)
Surgically altered anatomy	6 (35.3%)
Reason for bile duct stricture	
Malignancy	14 (82.3%)
Benign stenosis	3 (17.7%)
Etiology of stent occlusion	
Ingrowth	10 (58.8%)
Hyperplasia	7 (41.2%)
Reason for stent removal	
Anticipated long‐term survival	8 (46.9%)
Improvement of biliary stricture	3 (17.7%)
Stenting for benign biliary stricture	3 (17.7%)
Extensive bile duct stone formation	2 (11.8%)
Stent‐induced cholecystitis	1 (5.9%)
Cholecystitis	1 (7.7%)

Data are presented as *n* (%) or median (range).

### 3.2 Stent Characteristics

The characteristics of the stents are shown in Table 2. The types of first stent outer membranes were as follows: FCSEMS in 10 cases (58.8%), UCSEMS in four cases (23.5%), and partially covered SEMS (PCSEMS) in three cases (17.7%). The first stent was braided in 13 cases (76.5%) and laser‐cut in four cases (23.5%). The diameter of the first stent was 8 mm in 11 cases (64.7%) and 10 mm in six cases (35.3%). In all cases, the second stent had the same diameter as the first. The median duration from the first to the second stent placement was 17.4 months (range, 5.0–46.5 months). All secondary stents were FCSEMS, with the stent shape braided in 16 cases (94.1%) and laser‐cut in one case (5.9%). The median duration from the second stent placement to the attempt to remove the stent was 4.3 months (range, 0.3–12.3 months).

**TABLE 2:** Stent characteristics.

	*n* = 17
Type of first stent outer membrane	
Full covered	10 (58.8%)
Uncovered	4 (23.5%)
Partially covered	3 (17.7%)
Shape of the first stent	
Braided	13 (76.5%)
Laser cut	4 (23.5%)
Diameter of first stent	
8 mm	11 (64.7%)
10 mm	6 (35.3%)
Dwell time of first stent^†^ [months]	17.4 (5.0–46.5)
Type of second stent outer membrane	
Fully covered	17 (100%)
Shape of the second stent	
Braided	16 (94.1%)
Laser cut	1 (5.9%)
Diameter of the second stent	
8 mm	10 (58.8%)
10 mm	7 (41.2%)
Dwell time of second stent^‡^ [months]	4.3 (0.3–12.3)

Data are presented as *n* (%) or median (range).

^†^ Dwell time of the first stent: The period from the first to the second stent placement.

^‡^ Dwell time of second stent: The period from second stent placement to the stent removal procedure.

### 3.3 Clinical Outcome

**FIGURE 2:**
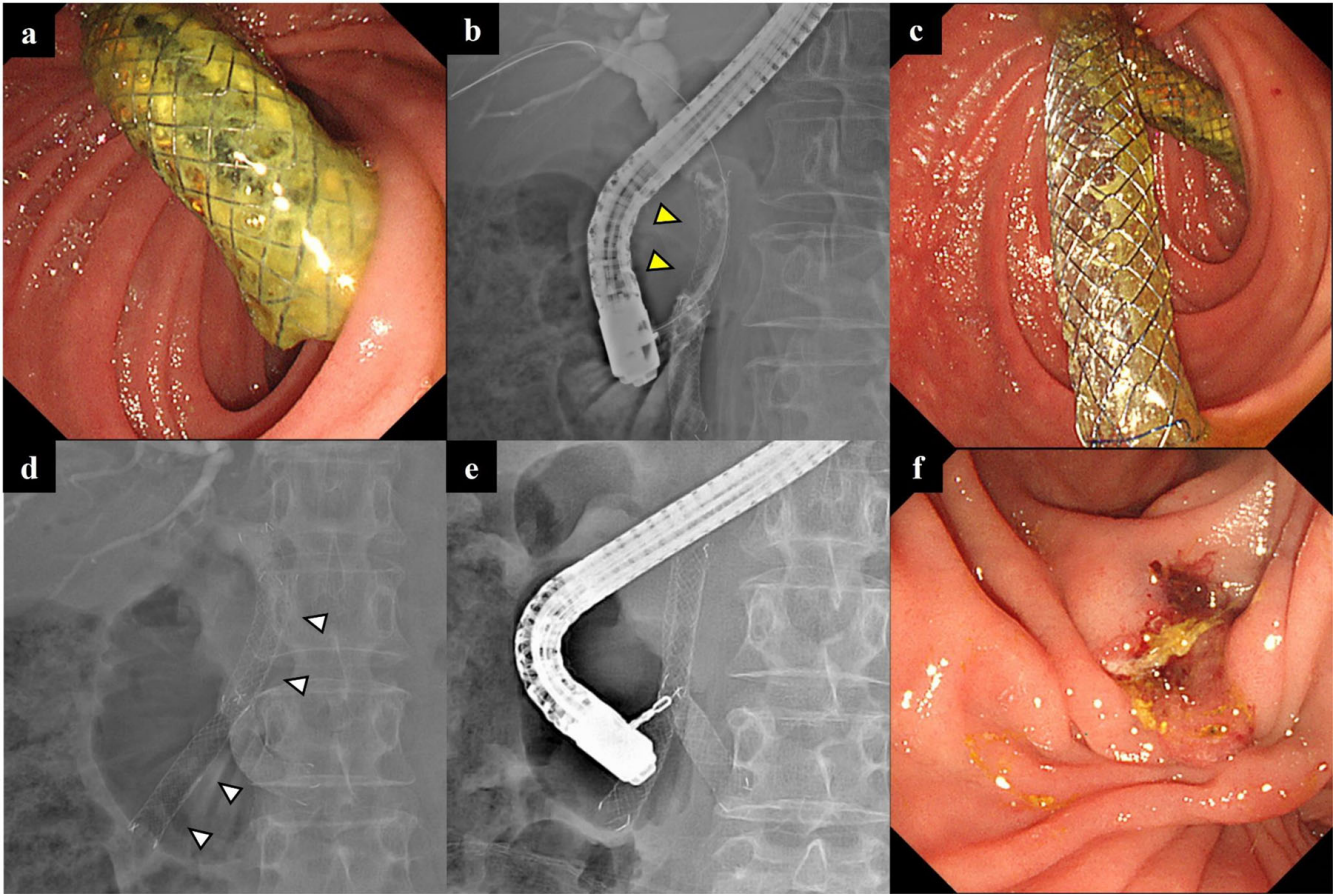
Stent‐in‐stent method via the transpapillary approach. (a) The fully covered self‐expandable metal stent that has been indwelling for 23.6 months becomes occluded. (b) The first stent has become embedded and cannot be removed (yellow arrowhead). (c, d) Using the stent‐in‐stent method, the second stent is placed through the mesh of the first stent (white arrowhead). (e, f) After 4.3 months, both stents are easily removed using grasping forceps.

**FIGURE 3:**
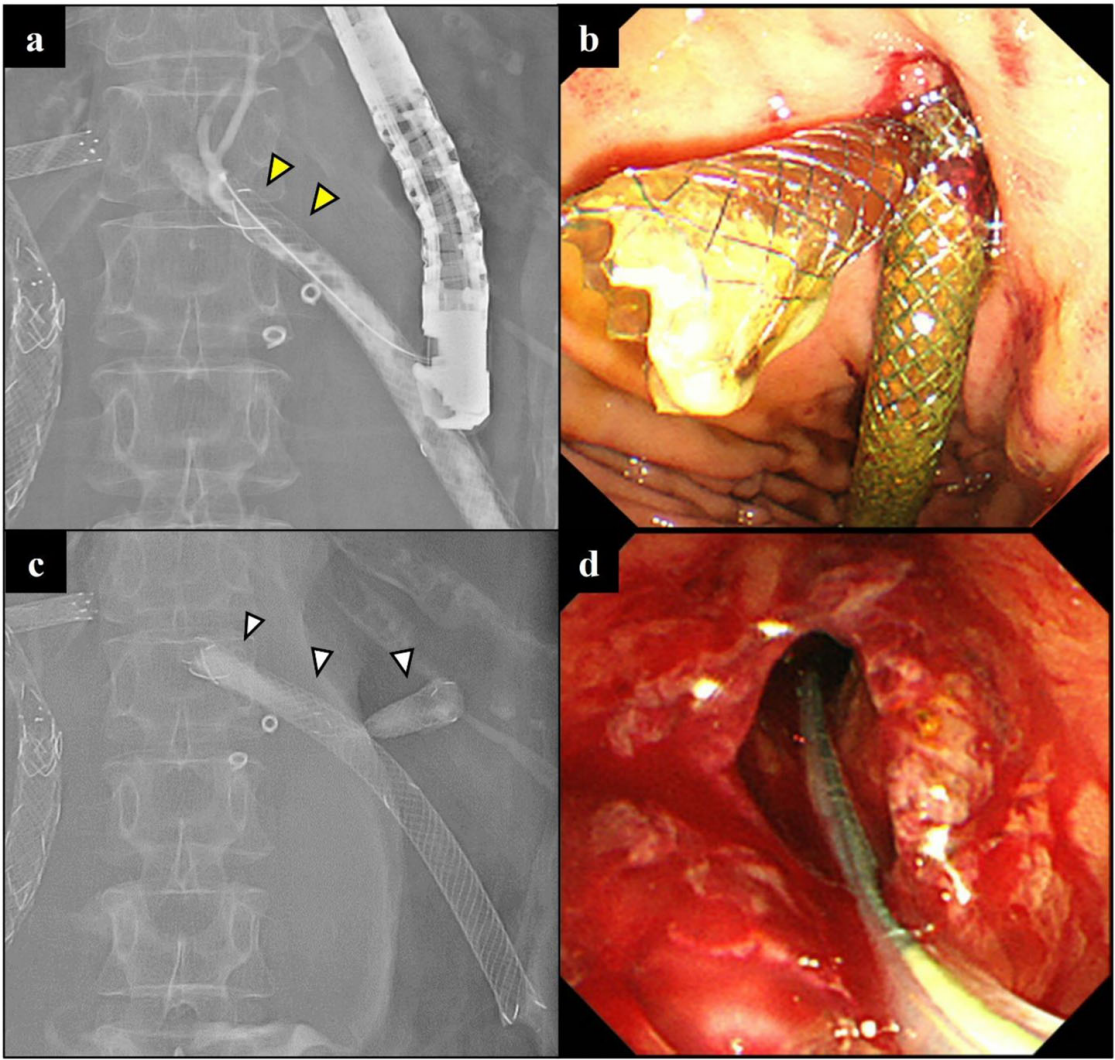
Stent‐in‐stent method via the endoscopic ultrasonography‐guided hepaticogastrostomy route. (a) Six months after the first stent placement, the stent tip has become embedded owing to hyperplasia, making removal impossible (yellow arrowhead). (b) The second stent is placed through the mesh of the first stent. (c) Fluoroscopic image after placing the second stent (white arrowhead). (d) Two months after the second stent placement, both stents are easily removed using grasping forceps.

The outcomes of the procedures are summarized in Table 3. The overall technical success rate of the SIS method for all the stents was 76.5% (13/17). The technical success rates according to route were 80.0% (8/10) for transpapillary cases (Figure 2), 100% (4/4) for EUS‐HGS (Figure 3), and 33.3% (1/3) for choledochojejunal anastomosis. The reasons for removal failure were as follows: stent fracture during attempted removal occurred in two transpapillary cases and one choledochojejunal anastomosis case, and removal of only the second stent occurred in one case of choledochojejunal anastomosis. The overall rate of adverse events associated with this procedure was 5.9% (1/17). Adverse events occurred in 9.1% (1/11) of the transpapillary cases, whereas no adverse events were observed in cases using EUS‐HGS or choledochojejunal anastomosis. One adverse event was cholecystitis following transpapillary placement of a second stent.

**TABLE 3:** Clinical outcomes.

	All cases (*n* = 17)	Transpapillary (*n* = 10)	EUS‐HGS route (*n* = 4)	Choledochojejunal Anastomosis (*n* = 3)
**Technical success rate, % (*n*)**	76.5% (13)	80.0% (8)	100% (4)	33.3% (1)
**Reasons for failure**				
** –Stent fracture, *n***	3	2	0	1
** –Second stent only removed, *n***	1	0	0	1
**Adverse event rate, % (*n*)**	5.9% (1)	9.1% (1)	0%	0%
**Details of the adverse event, *n***				
** –Cholecystitis, *n***	1	1	0	0

EUS‐HGS, endoscopic ultrasound‐guided hepaticogastrostomy.

Table 4 lists the details of each case. The SIS method enabled the removal of the first stents even when they were PCSEMS or UCSEMS. Patient No. 5 had an adverse event where cholecystitis occurred owing to cystic duct obstruction after the second stent placement. We attempted to remove both stents with grasping forceps 21 days after placing the second stent, but this was unsuccessful. However, the cholecystitis was resolved by slightly moving the stent distally in the bile duct. In Patient No. 15, an attempt to remove the embedded SEMS at the choledochojejunal anastomosis 9 days after placing a laser‐cut type FCSEMS inside its lumen was unsuccessful. Twenty‐one days after the second stent placement, both stents were successfully removed by pushing them into the jejunum with a balloon catheter through the percutaneous transhepatic biliary drainage route. The stents displaced into the jejunum were subsequently retrieved using a double‐balloon endoscope. Focusing on the etiology of stent embedding, the removal rate was 60% (6/10) for ingrowth and 100% (7/7) for hyperplasia.

**TABLE 4:** Patient and stent details.

			First stent						Second stent							
Pt	Stent location	Etiology	Name	Type		Diameter (mm)	Length (mm)	Dwell time (months)	Name	Type		Diameter (mm)	Length (mm)	Dwell time (months)	Technical success	Adverse event
1	TP	Ingrowth	BONASTENT	PC	Braided	10	80	14.7	WallFlex	FC	Braided	10	80	12.3	Yes	No
2	TP	Ingrowth	WallFlex	FC	Braided	10	60	31.0	BONASTENT	FC	Braided	10	60	10.8	Yes	No
3	TP	Ingrowth	unknown	UC	Braided	10	70	13.4	BONASTENT	FC	Braided	10	70	4.6	Yes	No
4	TP	Ingrowth	HILZO STENT	FC	Braided	10	60	17.4	BONASTENT	FC	Braided	10	60	1.7	Yes	No
5	TP	Ingrowth	BONASTENT	FC	Braided	10	70	15.9	BONASTENT	FC	Braided	10	80	0.7	**No**	**Yes**
6	TP	Ingrowth	BONASTENT	FC	Braided	8	80	23.6	BONASTENT	FC	Braided	8	80	4.3	Yes	No
7	TP	Ingrowth	BONASTENT	FC	Braided	8	60	28.1	BONASTENT	FC	Braided	8	80	8.7	Yes	No
8	TP	Ingrowth	BONASTENT	FC	Braided	8	80	14.1	BONASTENT	FC	Braided	8	80	8.3	**No**	No
9	TP	Hyperplasia	EGIS	FC	Braided	8	80	15.1	BONASTENT	FC	Braided	8	80	5.3	Yes	No
10	TP	Hyperplasia	EGIS	FC	Braided	10	60	44.1	BONASTENT	FC	Braided	10	80	6.2	Yes	No
11	HGS	Hyperplasia	X‐suit NIR	FC	Laser cut	8	80	46.5	BONASTENT	FC	Braided	8	80	2.1	Yes	No
12	HGS	Hyperplasia	Niti‐S	PC	Braided	8	120	7.0	BONASTENT	FC	Braided	8	60	2.0	Yes	No
13	HGS	Hyperplasia	HANAROSTENT	PC	Braided	8	120	17.1	HANAROSTENT	FC	Braided	8	100	2.7	Yes	No
14	HGS	Hyperplasia	X‐suit NIR	FC	Laser cut	8	80	38.2	BONASTENT	FC	Braided	8	100	8.1	Yes	No
15	CA	Ingrowth	BONASTENT	UC	Braided	8	60	5.0	Duckbill	FC	Laser cut	8	60	0.3	**No**	No
16	CA	Hyperplasia	Niti‐S	UC	Laser cut	8	40	34.4	BONASTENT	FC	Braided	8	30	3.2	Yes	No
17	CA	Ingrowth	Niti‐S	UC	Laser cut	8	80	30.6	BONASTENT	FC	Braided	8	70	0.8	**No**	No

Abbreviations: CA, choledochojejunal anastomosis; FC, fully covered; HGS, EUS‐guided hepaticogastrostomy; PC, partially covered; TP, transpapillary; UC, uncovered; BONASTENT, BONASTENT Biliary (Standard Sci Tech Inc., Seoul, South Korea); WallFlex, WallFlex (Boston Scientific Corp., Marlborough, MA, USA); HILZO STENT, HILZO STENT (BCM Co., Ltd, Paju, Korea); EGIS, EGIS Biliary Stent (S&G Biotech, Yongin, Korea), X‐suit NIR, X‐suit NIR (Olympus America Inc., Center Valley, PA, USA); HANAROSTENT, HANAROSTENT Biliary (M. I. Tech Co., Ltd., Pyeongtaek, Gyeonggi‐do, Korea); Niti‐S, Niti‐S large‐cell SR slim delivery system (Taewoong Medical, Gyeoenggi‐do, Korea); Duckbill, Duckbill Biliary Stent (Kawasumi Laboratories Inc., Tokyo, Japan)

Dwell time of the first stent: The period from the first to the second stent placement.

Dwell time of the second stent: The period from second stent placement to stent removal.

## 4 Discussion

### 4.1 Brief Summary

In this study, we attempted to remove 17 embedded biliary SEMS using the SIS method. The technical success rates for stent removal in the transpapillary, EUS‐HGS, and choledochojejunal anastomosis groups were 80.0%, 100%, and 33.3%, respectively. The overall technical success rate across all cases was 76.5%. The overall adverse event rate was 5.9%, with a single adverse event occurring in one patient with a transpapillary embedded stent. To the best of our knowledge, several studies have reported on the removal of stents via the SIS method for embedded biliary stents. However, all of these studies were limited to case reports involving five or fewer patients. Therefore, our study represents the largest case series on this technique currently available and is the first to evaluate the utility of the SIS method not only in transpapillary cases but also via EUS‐HGS and in patients with choledochojejunal anastomosis.

### 4.2 Previous Reports on the SIS Method

Table 5 summarizes previously reported cases of biliary‐embedded stent removal using the SIS method [7, 10, 11, 12, 13, 14, 15, 16, 17, 18]. The technical success rate was 85.7% (12/14), with no reported adverse events. Two failed removals occurred: one used a second uncovered stent [15], and the other had a laser‐cut first stent [17]. In these two cases, an additional stent allowed for the removal of all three later. All cases used a second stent of equal or greater diameter than the first, which is appropriate given that the SIS method compresses the tissue within the stent.

**TABLE 5:** Previous reports on the stent‐in‐stent technique for the removal of embedded biliary stents

				First stent				Second stent				
Author	*n*	Etiology	Stent location	Type		Diameter (mm)	Dwell time (months)	Type		Diameter (mm)	Dwell time (months)	Technical success
Arias D., 2010 [5]	1	Hyperplasia	TP	UC	Braided	10	12.0	FC	Braided	N/A	1.0	Yes
Tan D. M., 2012 [8]	1	Hyperplasia	TP	UC	Braided	N/A	1.5	FC	Braided	10	0.5	Yes
Menon S., 2013 [9]	1	Hyperplasia	TP	FC	Braided	10	5.0	FC	Braided	10	1.5	Yes
Gonzalez N., 2013 [10]	1	Hyperplasia	TP	UC	N/A	N/A	2.1	FC	N/A	N/A	0.3	Yes
Tringali A., 2014 [11]	5	Hyperplasia	TP	FC	Braided	10	11.0^†^	FC	Braided	10	1.0^†^	Yes
Mangira D., 2014 [12]	1	Hyperplasia	TP	FC	Braided	10	14.0	FC	Braided	10	0.5	Yes
Yu T., 2022 [13]	1	Hyperplasia	TP	UC	Braided	8	120.0	UC	Braided	8	36.0	No
Matsumi A., 2022 [14]	1	Hyperplasia	TP	FC	Braided	N/A	120.0	FC	Braided	N/A	1.0	No
Matsuyama M., 2023 [15]	1	Ingrowth	TP	FC	Laser cut	10	15.0	FC	Braided	N/A	1.0	Yes
Ogura T., 2023 [16]	1	Hyperplasia	HGS	PC	Braided	8	3.0	FC	Braided	8	0.2	Yes
(Our study)	17	Various situations										76.5%

Abbreviations: FC, fully covered; HGS, EUS‐guided hepaticogastrostomy; N/A, not available; PC, partially covered; TP, transpapillary; UC, uncovered.

Dwell time of the first stent: The period from the first to the second stent placement.

Dwell time of the second stent: the period from the second stent placement to the stent removal trial.

^†^ The median periods of the five cases are presented.

### 4.3 Optimal Timing for Stent Removal

Regarding the timing of stent removal, the median interval from the second stent placement to removal was 0.63 months (range 0.2–1.5) in previous reports, which was shorter than in our study (4.2 months). Based on five cases and prior reports, Tringali et al. suggested removing the second stent within 4 weeks, as prolonged placement may reduce compression and hinder removal [13]. In our study, 14 stent removals performed more than 4 weeks after the second stent placement were successful in 13 cases, corresponding to a technical success rate of 92.9%. In contrast, of the four cases in which stent removal failed, three had attempted removal within 4 weeks. Prior reports have also described difficulties when stent removal is attempted 10 days after the second stent placement [12]. This indicates that a certain period is required after the second stent placement to achieve sufficient tissue compression within the stent lumen. Based on our findings, we suggest that the timing of stent removal should exceed 4 weeks after the second stent placement.

### 4.4 Embedded Stents in EUS‐HGS or the Choledochojejunal Anastomosis

Most reports on the SIS method involve transpapillary stents, with only one case involving EUS‐HGS [18]. EUS‐HGS often uses a PCSEMS with an uncovered distal end to prevent migration and branch obstruction. Although this can cause hyperplasia and complicate removal, all four EUS‐HGS cases were performed smoothly without complications in our study. The SIS method appears effective and safe for use with embedded stents in EUS‐HGS. In contrast, the success rate of stent removal using the SIS method for embedded stents at the choledochojejunal anastomosis was low (33.3% [1/3]). In this anatomical setting, the distance between the anastomosis and confluence of the right and left hepatic ducts is short; therefore, UCSEMS are generally preferred to avoid occlusion of either duct. In our study, all the first stents placed at the choledochojejunal anastomosis were UCSEMS, and two of them were of the laser‐cut type. Because stent removal is often not anticipated at the time of placement in this setting, UCSEMS and laser‐cut stents, which are more difficult to remove, are more likely to be selected. Additionally, procedures involving choledochojejunal anastomosis require the use of a colonoscope or balloon‐assisted endoscope, which are technically more challenging than procedures using a duodenoscope. Therefore, the SIS method may not be effective for choledochojejunal anastomosis. Nevertheless, given the small sample size of only three cases in this study, further evaluation in larger cohorts is warranted.

### 4.5 Etiology of Embedded Stents

Our study included 10 cases of stent embedding caused by the ingrowth of a malignant tumor. In previous reports, most cases of stent embedding were for benign strictures, with only one reported case of successful stent removal involving malignant stenosis [17]. Embedded stents in benign strictures are caused by hyperplasia of the benign tissue, whereas those in malignant strictures result from tumor ingrowth into the stent, which makes their removal more challenging. In our study, the success rate of stent removal using the SIS method was 100% (7/7) in patients with benign hyperplasia and 60.0% (6/10) in those with malignant ingrowth. Although the SIS method is expected to be effective in displacing hyperplastic benign tissues, its efficacy may be limited to malignant tissues owing to their firmer consistency.

### 4.6 Proposal of a Strategy for the SIS Method

In the cases where stent removal failed, the tissue that had proliferated into the lumen of the first stent acted as an anchor, and when traction was applied, the stent was damaged without being removed. The SIS method is a technique that utilizes the compressive effect of the second stent on the ingrown tissue; however, in cases where stent removal was unsuccessful, it is considered that the tissue extending into the first stent was not sufficiently compressed by the second stent.

From the present study, it was suggested that in cases where difficult removal is anticipated—such as embedded stents placed at the choledochojejunal anastomosis or stent embedding due to malignant tumor ingrowth—attempting removal at least four weeks after the placement of the second stent, when sufficient tissue compression has been achieved, may contribute to successful removal. Moreover, although this point was not sufficiently examined in this study, in cases of difficult removal, the use of a second stent with a larger diameter or stronger radial force may help improve the success rate of stent removal by achieving greater tissue compression.

### 4.7 Limitations

Our study has several limitations, including the small sample size, retrospective design, and the fact that it was conducted at a single center. Future studies with larger cohorts, particularly those that include patients who underwent EUS‐BD or choledochojejunal anastomosis, are warranted. Moreover, this study included cases in which removal of the embedded stent was not initially planned at the time of the second stent placement.

## 5 Conclusion

Stent removal using the SIS method for embedded stents appears to be effective not only via the transpapillary route but also via EUS‐HGS. However, it may not be suitable for cases involving choledochojejunal anastomoses. Delayed removal beyond 4 weeks may improve technical success. The effectiveness of the SIS method may be limited in cases of stent embedding due to malignant tissue ingrowth.

## Conflicts of Interest

Nao Fujimori is an Associate Editor of DEN Open. The other authors declare no conflicts of interest.

## Funding

This work was supported by the National Cancer Center Research and Development Fund [grant number 2022‐A‐16].

## Ethics Statement

**Approval of the Research Protocol by an Institutional Reviewer Board**: This study was conducted in accordance with the principles of the Declaration of Helsinki and approved by the Ethics Committee of the National Cancer Center Hospital, Japan (Ethical Approval Number: 2018‐149).

## Consent

N/A

## Registry and the Registration No. of the Study/Trial

N/A

## Supporting information




**FIGURE S1**: (a) Tissue extension into the lumen of the biliary stent with stent embedding. (b) The second stent shows incomplete coverage of the tissue extending into the stent lumen. (c) The second stent completely covers the tissue extending into the stent lumen. This condition is referred to as the stent‐in‐stent method.

**VIDEO S1**: Stent‐in‐stent methods for embedding stents in various situations.
