# Supplementary figures and images for: Efficacy of the Stent‐in‐Stent Technique as a Rescue Method for Removing Embedded Metallic Biliary Stents
Source: DEN Open. 2025 Dec 17;6(1):e70246. doi: 10.1002/deo2.70246 (PMC12710792; doi:10.1002/deo2.70246)

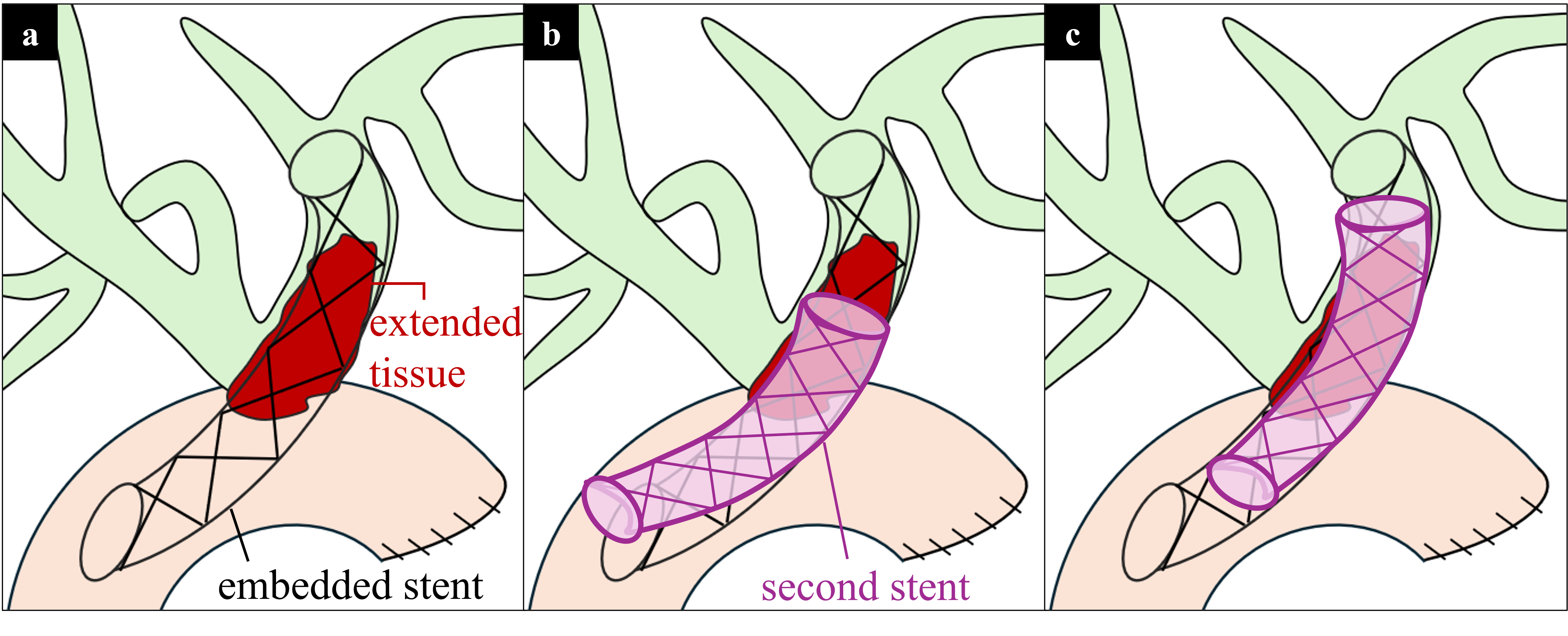

Supplement: Supplementary file 1 — FIGURE S1: (a) Tissue extension into the lumen of the biliary stent with stent embedding. (b) The second stent shows incomplete coverage of the tissue extending into the stent lumen. (c) The second stent completely covers the tissue extending into the stent lumen. This condition is referred to as the stent‐in‐stent method. [file DEO2-6-e70246-s002.png]
